# AMP-Activated Protein Kinase and O-GlcNAcylation, Two Partners Tightly Connected to Regulate Key Cellular Processes

**DOI:** 10.3389/fendo.2018.00519

**Published:** 2018-09-13

**Authors:** Roselle Gélinas, Justine Dontaine, Sandrine Horman, Christophe Beauloye, Laurent Bultot, Luc Bertrand

**Affiliations:** ^1^Montreal Heart Institute, Université de Montreal, Montreal, QC, Canada; ^2^Institut de Recherche Expérimentale et Clinique, Pole of Cardiovascular Research, Université catholique de Louvain, Brussels, Belgium; ^3^Division of Cardiology, Cliniques Universitaires Saint-Luc, Brussels, Belgium

**Keywords:** AMPK, O-GlcNAcylation, diabetes, cardiovascular diseases, cancer

## Abstract

The AMP-activated protein kinase (AMPK) is an important cellular energy sensor. Its activation under energetic stress is known to activate energy-producing pathways and to inactivate energy-consuming pathways, promoting ATP preservation and cell survival. AMPK has been shown to play protective role in many pathophysiological processes including cardiovascular diseases, diabetes, and cancer. Its action is multi-faceted and comprises short-term regulation of enzymes by direct phosphorylation as well as long-term adaptation via control of transcription factors and cellular events such as autophagy. During the last decade, several studies underline the particular importance of the interaction between AMPK and the post-translational modification called O-GlcNAcylation. O-GlcNAcylation means the O-linked attachment of a single N-acetylglucosamine moiety on serine or threonine residues. O-GlcNAcylation plays a role in multiple physiological cellular processes but is also associated with the development of various diseases. The first goal of the present review is to present the tight molecular relationship between AMPK and enzymes regulating O-GlcNAcylation. We then draw the attention of the reader on the putative importance of this interaction in different pathophysiological events.

## Introduction

The majority of proteins playing essential biological roles undergo post-translational modification (PTM) to regulate their structure, cellular localization, activity and biological function. Accordingly, many metabolic enzymes and regulators, such as acetyl-CoA carboxylase and ULK1, are known to be phosphorylated in conditions of metabolic stress to adapt to energy imbalance (1). Under such circumstances, cells will adjust their metabolism by promoting energy production pathways and shutting down non-essential energy-consuming pathways. An essential actor in the phospho-dependent metabolic reorganization during energy stress is the AMP-activated protein kinase (AMPK) (2). Similar to protein phosphorylation, O-linked β-N-acetylglucosamine (O-GlcNAc) addition on Ser/Thr residues is a dynamic PTM that regulates many cellular processes including stress response (3, 4), transcriptional activity (5, 6), and epigenetic regulation (7). It has been recently shown that AMPK can interrelate with enzymes regulating protein O-GlcNAcylation. The present review highlights this interplay with an emphasis of how this link could be targeted to prevent and/or treat several diseases.

### AMPK, a key energy sensor

AMPK is a heterotrimeric protein composed of one catalytic subunit α (existing in two isoforms, α1, and α2) and two regulatory subunits, β (β1 and β2) and γ (γ1, γ2, and γ3) (8). AMPK is a vital cellular energy sensor that regulates metabolism in order to maintain energy balance (9). During energy stress, the increase in AMP/ATP ratio leads to the binding of AMP to the γ subunit promoting AMPK activation by allosteric regulation and by phosphorylation of the catalytic α subunit on Thr172 (10). Once activated, AMPK phosphorylates downstream targets at Ser/Thr residues within a characteristic sequence motif. Numerous AMPK substrates in various protein networks have been described so far (1, 11, 12). AMPK acts acutely by phosphorylating metabolic enzymes, but also provokes long-term adaptation at a transcriptional level (13). AMPK inhibits anabolic pathways, including protein synthesis (14, 15), and enhances catabolic pathways, such as glycolysis and mitochondrial β-oxidation, to restore energetic balance required for cell survival (16, 17). AMPK activation is considered as a putative future therapeutic target for various pathologies characterized by disorganized cellular metabolism, such as cancer, diabetes, myocardial ischemia and cardiac hypertrophy (18). In order to develop new drugs and treatments, deciphering the mechanisms by which AMPK provides its beneficial action have pushed research in the field. Beside AMPK, a significant proportion of these diseases is also characterized by an atypical O-GlcNAcylation profile. Recent evidences demonstrate interplay between AMPK and O-GlcNAcylation. Interestingly, some of the AMPK beneficial effects seem to be linked to modulation of O-GlcNAcylation.

### O-GlcNAcylation, a particular post-translational modification

**Figure 1:**
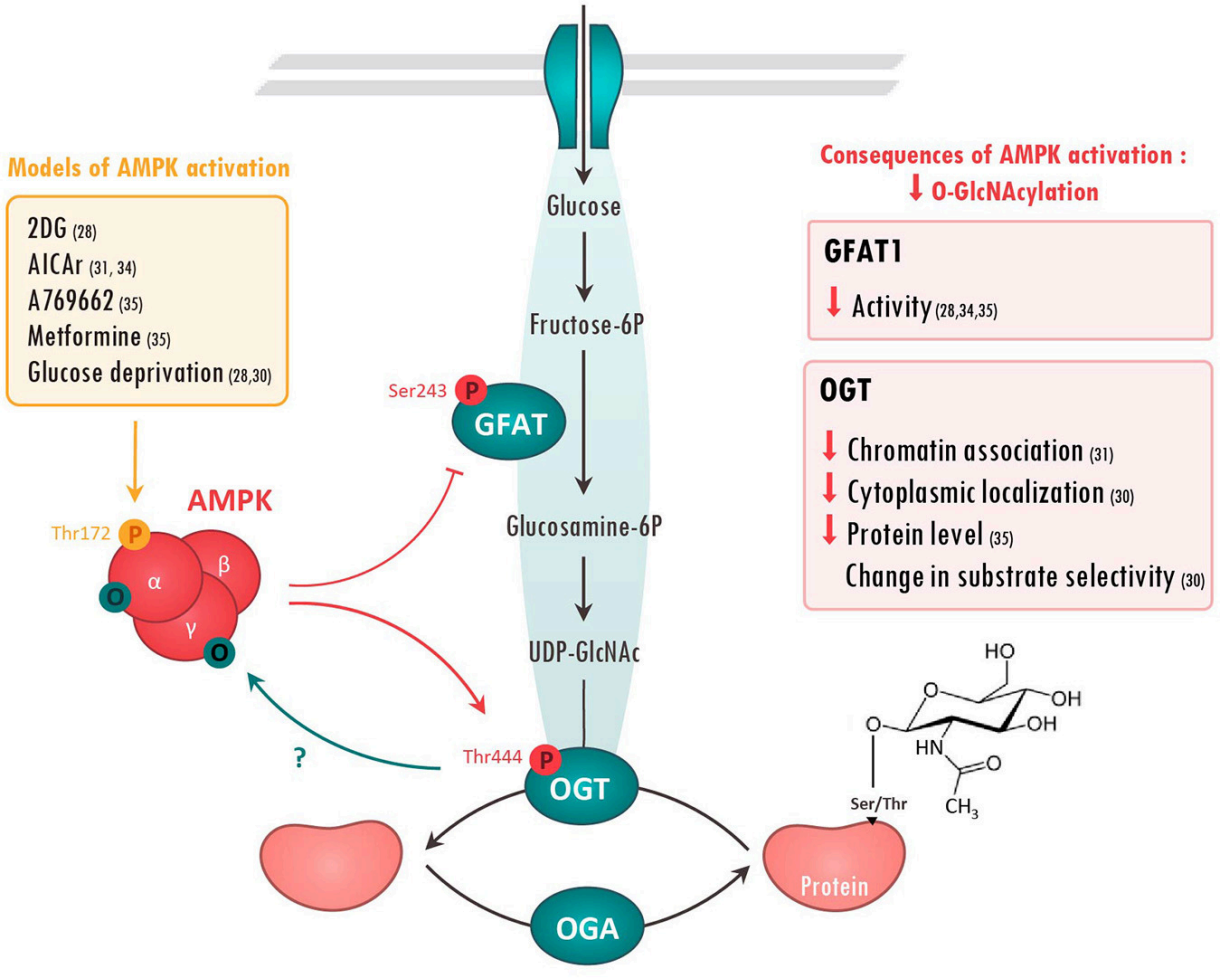
Molecular interactions between AMPK and O-GlcNAcylation-mediating enzymes. Action of AMPK on O-GlcNAcylation-mediating enzymes has been evaluated in the literature using various treatments (left panel). Once activated, AMPK has been shown to phosphorylate Glutamine fructose-6-phosphate aminotransferase (GFAT) on Ser243 and O-GlcNAc transferase (OGT) on Thr444. The consequences of these AMPK-mediated modifications are resumed in the right panels. On the other hand, AMPK can be O-GlcNAcylated on its α and γ subunits, presumably preventing its activation. References are presented in parentheses. 2DG, 2 Deoxyglucose; AICAr, 5 aminoimidazole-4-carboxamide ribonucleoside; O, O-GlcNAcyl; P, phosphoryl group; UDP-Glucose, Uridine diphosphate glucose.

Protein O-GlcNAcylation is a PTM that promotes the O-linked attachment of a single N-acetylglucosamine moiety on Ser/Thr residues of over 4000 proteins including nuclear, cytosolic, and mitochondrial proteins (5). O-GlcNAcylation depends on the availability of uridine diphosphate-N-acetylglucosamine (UDP-GlcNAc), the end product of hexosamine biosynthesis pathway (HBP), an alternative pathway of glucose metabolism (Figure 1). HBP is dependent of glucose, amino acid and nucleotide availability, turning O-GlcNAcylation to a nutrient sensitive PTM that dynamically integrates metabolic signals (19, 20). The increased HBP flux due to chronic metabolic changes gives rise to elevated levels of protein O-GlcNAcylation which are known to play a role in the development of insulin resistance and diabetes (21, 22). Beyond diabetes, aberrant O-GlcNAcylation is associated with several diseases, such as cardiovascular diseases and cancer. Glutamine:fructose-6-phosphate aminotransferase (GFAT), which converts fructose 6-phosphate to glucosamine 6-phosphate, is the rate limiting enzyme of HBP and its deletion causes vital cellular defects (23). GFAT is highly conserved among species and two isoforms from separated genes, GFAT1 and GFAT2, can be found in mammals (24). Their relative expression varies among tissues, GFAT1 being mainly expressed in pancreas, placenta, testis and skeletal muscle whereas GFAT2 can be found in heart and central nervous system (25).

Unlike phosphorylation, which is regulated by numerous protein kinases and phosphatases, O-GlcNAc cycling is regulated by the concerted action of only two enzymes, O-GlcNAc transferase (OGT) that adds *O*-GlcNAc to proteins, and the protein O-GlcNAcase (OGA) that removes it (26) (Figure 1). This limited number of actors has suggested that O-GlcNAcylation was an unrefined modification hitting non-specifically a large number of substrates. However, recent evidences show that OGT/OGA localization, their interactions with specific substrates and the competition for Ser/Thr with other PTMs shape the O-GlcNAcylation response in agreement with cellular state. As an example, competition between phosphorylation and O-GlcNAc for the same or proximal site is well documented in the recent review of van der Laarse et al. (27).

Growing evidences indicate that both GFAT and OGT can be substrates of protein kinases including AMPK (28, 29). Conversely, AMPK can be targeted by O-GlcNAcylation (30, 31). The next chapters will present the molecular basis of these molecular interactions and the putative consequences of this interplay in pathologies.

## Crosstalk between AMPK and O-GlcNAcylation-mediating enzymes

### Regulation of O-GlcNAcylation by AMPK

#### GFAT

Regulation of O-GlcNAc by AMPK has been firstly described in 2007 when AMPK was proposed to phosphorylate GFAT1 on Ser243 (32) (Figure 1). This phosphorylation was postulated to increase GFAT activity by lowering *k*_*M*_ for fructose-6-phosphate as measured by *in vitro* enzymatic assay. However, more recent studies rather demonstrate that AMPK negatively regulates GFAT activity. Using model of GFAT1 overexpression in mammalian cells, Eguchi and colleagues reported that 2-deoxyglucose-mediated AMPK activation induces GFAT1 phosphorylation on Ser243, concomitantly to its inactivation (28). Similarly, AMPK activation, by 5-aminoimidazole-4-carboxamide riboside (AICAr) or vascular endothelial growth factor (VEGF) in primary human endothelial cells, has been shown to induce a marked increase in endogenous GFAT1 phosphorylation on Ser243, a significant reduction in GFAT activity and a concomitant reduction in O-GlcNAc levels (33, 34). Finally, our research group has shown that AMPK activation by metformin, or by the specific AMPK activator A769662, promotes GFAT phosphorylation on the aforementioned site in both neonatal rat cardiomyocytes (NRVM) and adult mouse heart (35) Once again, this is associated with reduced O-GlcNAc levels (35). Overall, majority of the recent studies demonstrate that AMPK directly phosphorylates and reduces GFAT activity, lowering O-GlcNAc levels (Figure 1).

#### OGT

Besides GFAT, OGT also looks to be regulated by AMPK (Figure 1). It has been shown that AMPK can interact with and phosphorylate OGT on Thr444 *in vitro* (30). Moreover, AMPK activation by A769662 or AICAr triggers OGT phosphorylation on Thr444 in skeletal muscle cells and mouse embryonic fibroblasts (MEF) (30, 31). Interestingly, Thr444 phosphorylation does not affect OGT enzymatic activity, but rather seems to modulate OGT cellular localization and substrate specificity. Xu and colleagues revealed that AMPK phosphorylates nuclear OGT in MEFs, promoting its dissociation from chromatin and the reduction of histone 2B O-GlcNAcylation on Ser112 (31). By contrast, AMPK activation is shown to induce OGT nuclear translocation in C2C12 myotubes, stimulating nuclear protein O-GlcNAcylation and histone acetylation (30). In addition to modulating cellular localization, AMPK seems to alter OGT substrate selectivity by promoting its interaction with particular proteins or by competing with OGT for the same or proximal site(s). As example, AMPK decreases 26S proteasome activity in endothelial cells by promoting its association with OGT and its subsequent O-GlcNAcylation (36). Moreover, competition between O-GlcNAcylation and AMPK phosphorylation for the same or proximal Ser/Thr site is described to have opposite effects on the regulation of several proteins. For example, AMPK phosphorylation and O-GlcNAcylation compete for the same site on chromatin-associated fumarase (FH) and lead to opposite effects (37). Hyaluroran HAS2 activity is also regulated in an opposite manner by AMPK phosphorylation and O-GlcNAcylation on proximal sites, although competition between both is not yet formerly studied (38).

AMPK has also been proposed to regulate OGT expression. In Neuro-2a neuroblastoma cells, glucose deprivation induces an AMPK-dependent increase in OGT mRNA and protein expression, leading to elevated O-GlcNAc levels (39). However, two other studies, performed in other cell types, established that glucose deprivation acts on OGT independently of AMPK (40, 41). In our hands, we showed that metformin-induced AMPK activation leads to a decrease in cardiac OGT expression and O-GlcNAc levels in hypertrophied mouse hearts. In conclusion, the regulation of OGT expression by AMPK seems to highly depend of various parameters such as cell types and physio-pathological status.

#### OGA

Evidence of direct regulation of OGA by AMPK has not been demonstrated so far. However, mice deficient for the AMPKα2 isoform are characterized by an elevated basal level of cardiac O-GlcNAcylation that is associated with decreased OGA protein levels (35). The molecular mechanism involved remains to be identified.

### Regulation of AMPK by O-GlcNAcylation

In a reciprocal way, AMPK can be regulated by O-GlcNAcylation (Figure 1). The first evidence dates back to 2007. AMPK was detected in O-GlcNAc precipitates using succinylated wheat germ agglutinin, a lectin that binds O-GlcNAc residues (42). In 2014, two different research groups confirmed that AMPK can be O-GlcNAcylated by OGT. Xu et al. report that AMPKα1 isoform is O-GlcNAcylated *in vitro* in MEFs and that OGT knock-down reduces AMPK phosphorylation on Thr172, suggesting that OGT positively regulates AMPK activity (31). With the same idea, Bullen and collaborators showed that all AMPKα- and γ-subunits are “O-GlcNAcylable” *in vitro* (30). They also demonstrated that O-GlcNAcylation of an over-expressed form of AMPKγ1 subunit occurs in human embryonic kidney (HEK) 293 cells. Interestingly, this O-GlcNAcylation happens when AMPK is activated by glucose deprivation or AICAr treatment and is absent when a kinase dead form of AMPK is over-expressed. This highly suggests that AMPK activation is required for its own O-GlcNAcylation. However, it has to be mentioned that AMPK/O-GlcNAcylation interplay is extremely complex. Indeed, pre-treatment with O-GlcNAc inducers, such as Thiamet G, blunts AMPK activation induced by glucose deprivation, indicating that PTM order plays an important role in AMPK regulation by O-GlcNAcylation (30). Accordingly, EtOH-induced increase in AMPK phosphorylation on Thr172 coincides with a decrease in AMPK O-GlcNAcylation in C2C12 myocytes (43). Other studies report alternatives effects of O-GlcNAcylation on AMPK activity. Laczy and colleagues reported no changes in AMPK activation in isolated rat hearts perfused with glucosamine (44). In the same line, our group established that increasing O-GlcNAc levels using O-GlcNAc inducers or decreasing O-GlcNAcylation with the GFAT inhibitor 6-diazo-5-oxo-L-norleucine (DON), have no impact on AMPK activity in neonatal rat cardiomyocytes and mouse hearts (35).

Besides direct regulation of AMPK by OGT, O-GlcNAcylation may induce AMPK-dependent phosphorylation of specific proteins. As example, ULK1 O-GlcNAcylation promotes AMPK recruitment and AMPK-mediated phosphorylation of ULK1, resulting in increased cellular autophagy (29).

As conclusion of this chapter, molecular crosstalk and/or competition between AMPK and O-GlcNAc pathway is well-documented. This interplay has distinct consequence depending on cell type, chronic/acute stimulation and metabolic status.

## Pathophysiological consequences of AMPK/O-GlcNAcylation interaction

**Figure 2:**
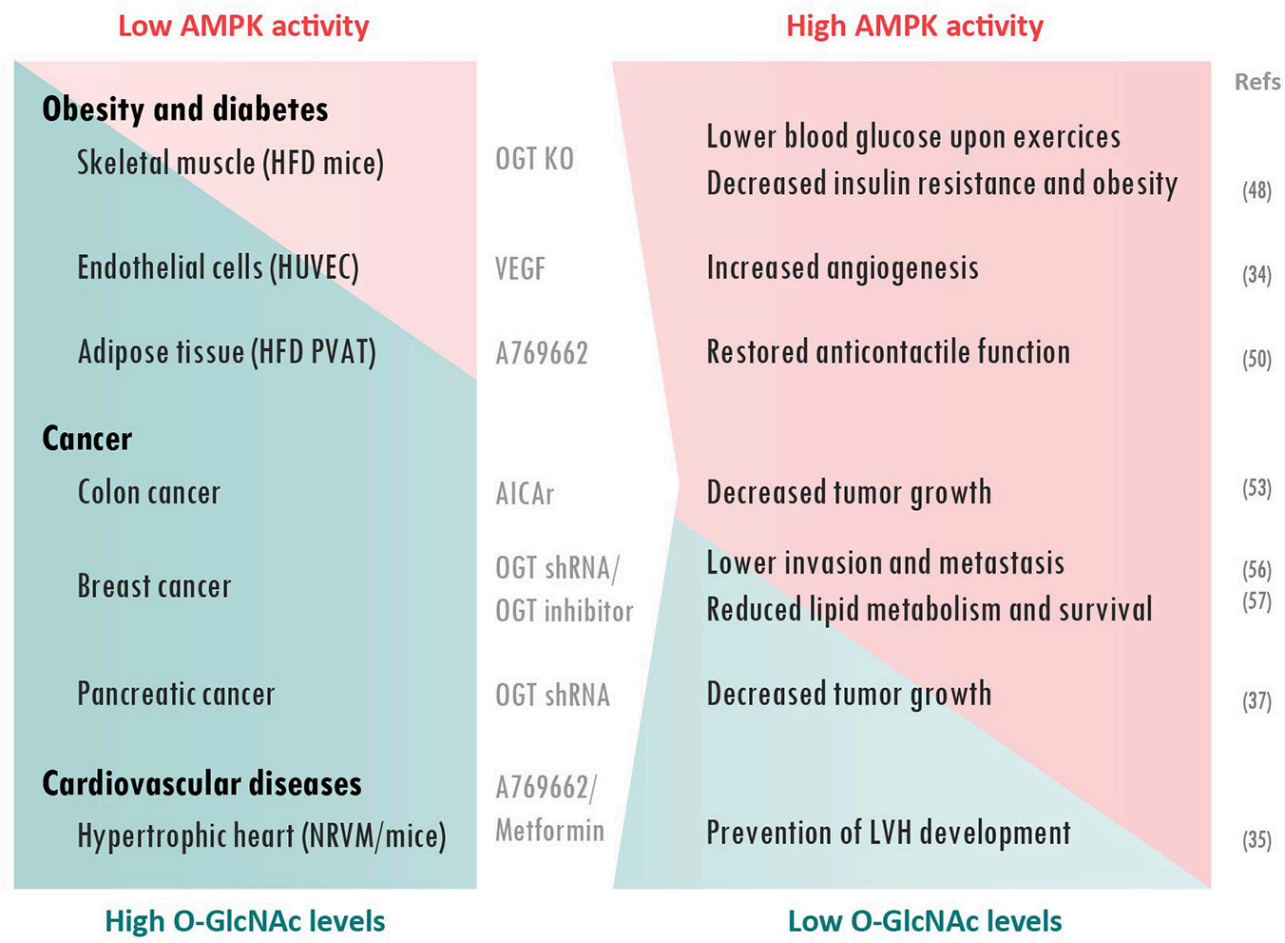
AMPK/O-GlcNAcylation interplay in pathophysiological processes. AMPK activation and O-GlcNAcylation levels are generally inversely correlated in pathologies. Lowering O-GlcNAcylation or increasing AMPK activity prevents adverse effects in diabetes, cancer and cardiac hypertrophy. Treatments and cellular/animal models used are presented between to two columns. AICAr, 5-aminoimidazole-4-carboxamide ribonucleotise; HFD, High-fat diet; HUVEC, Human Umbilical Vein Endothelial Cells; LVH, Left ventricular hypertrophy; NRVM, Neonatal rat ventricular myocytes; OGT KO, O-GlcNAc transferase knock-out; PVAT, Perivascular adipose tissue; shRNA, short hairpin RNA; VEGF, Vascular endothelial growth factor.

AMPK signaling and O-GlcNAcylation are associated with various pathologies such as diabetes, cancers and cardiovascular diseases (Figure 2). A global rise of O-GlcNAcylation is detected during the developement of these pathologies. Conversely, AMPK activation is generally described to be beneficial on slowing down their development. In this last part, we will overview how O-GlcNAcylation/AMPK interaction plays a role in these pathological events.

### Obesity and diabetes

During the last 30 years, number of studies provided direct evidences supporting a link between HBP and adverse effects of diabetes [reviewed in (45)]. As nicely reviewed by Copeland and collaborators, elevated protein O-GlcNAcylation is strongly associated with hyperglycemia-induced glucotoxicity and insulin resistance (46). On the other hand, AMPK is described to be beneficial for diabetic patients via a multi-faceted action including an insulin-independent stimulation of glucose transport (47). In addition, interaction between AMPK and O-GlcNAcylation has been highlighted in diabetic conditions. Indeed, it has been recently reported that skeletal muscle-specific OGT knock-out mice show lower blood glucose during exercise and reduced high-fat-mediated obesity and insulin resistance (48). Interestingly, these effects are associated with higher AMPK expression and greater AMPK activation after exercise. It has been proposed by the authors, that the reduction in muscle O-GlcNAcylation has an anti-diabetic effect through an enhancement of the AMPK-dependent glucose utilization during exercise (48).

Vascular complications such as endothelial dysfunction are another important event in type II diabetes. Elevated protein O-GlcNAcylation levels are known to be part of hyperglycaemia-induced inhibition of angiogenesis (49). It has been shown that VEGF promotes angiogenesis in endothelial cells through an AMPK-mediated decrease in protein O-GlcNAcylation (34). AMPK/O-GlcNAcylation was thus proposed as putative future therapeutic target to improve vascular dysfunction in diabetic patients.

Finally, O-GlcNAc and AMPK have also been proposed to be associated with prenatal programming of perivascular adipose tissue (PVAT) associated with obesity-linked hypertension. Indeed, PVAT from male offspring of rodent fed with an obesogenic high-fat diet (HFD) are characterized by reduced anti-contractile effect, elevated O-GlcNAc levels and low AMPK activity (50). Interestingly, incubation of PVAT from male control offspring rat with glucosamine, as O-GlcNAc inducer, reduced AMPK activity and diminished its anti-contractile properties. However, PVAT function was restored by simultaneous AMPK activation, using A769662. Similarly, AMPK activation partially restored anti-contractile effects of PVAT from HFD offspring. Overall, these results support the idea that elevated O-GlcNAcylation levels, seen in obesity are related to reduced AMPK activity and, concomitantly, loss of anti-contractile effect of PVAT.

### Cancer

Elevated protein O-GlcNAcylation has been reported in various cancer cells, including prostate, colon, breast and lung cancer as well as chronic myeloid leukemia (51) whereas AMPK has potent anti-tumoral properties (52). Furthermore, increasing O-GlcNAc levels, using Thiamet G or OGT overexpression, leads to an increase in AMPK O-GlcNAcylation and reduced AMPK activity in human colon cancer cells (53). This suppresses the inhibitory action of AMPK on the mammalian target of rapamycin pathway and results in enhanced tumor growth. Consequently, it is suggested that AMPK inactivation, due to elevated O-GlcNAc levels in diabetic patients, could explain the higher risk of colon cancer in this population (54).

Similar data were obtained in breast cancer cell lines by the group of Reginato (55–57). Increasing O-GlcNAcylation using NButGT or overexpression of OGT decreases AMPK activity, while reducing O-GlcNAcylation with OGT knock-down or OGT inhibitor leads to an increase in AMPK phosphorylation. In these studies, AMPK and OGT seem to have opposite effects on various proteins involved in cancer metabolism, such as the transcriptional regulator hypoxia-inducible factor-1α (55) and the deacetylase sirtuin 1 (SIRT1) (56). Among others, they showed that reducing O-GlcNAcylation increases SIRT1 protein levels in an AMPK-dependent manner; promoting degradation of oncogenic transcription factor forkhead box M1 in association with reduced invasion and metastasis of breast cancer. They also demonstrated that O-GlcNAcylation controls lipid metabolism in tumor cells via an AMPK-dependent mechanism. More precisely, inactivation of AMPK by OGT was shown to regulate sterol regulatory element-binding protein-1 phosphorylation and stability, resulting in higher lipid synthesis and, subsequently, in elevated cancer cell growth and survival (57).

Lastly, AMPK and O-GlcNAcylation can compete for a same Ser75 regulatory site of chromatin-associated FH involved in tumorigenesis. This gives rise to opposite effects of FH on the regulation of histone methylation (37). The increase in FH O-GlcNAcylation promotes development of pancreatic tumors (37). Consequently, the decrease of FH phosphorylation correlates with elevated protein O-GlcNAcylation and poor prognosis in pancreatic cancer patients.

### Cardiovascular diseases

An increase in global cardiac protein O-GlcNAcylation is commonly observed in pathological cardiac hypertrophy and heart failure, although the exact mechanism is not fully understood (58–60). AMPK, which is activated during left ventricular hypertrophy, acts as counter-regulatory mechanism and is proved to be largely protective (17). In a recent study, our research group established that the anti-hypertrophic action of AMPK is mainly explained by its inhibitory action on HBP via GFAT phosphorylation (35).

## Conclusion

Various observations raise evidences that AMPK activation reduces O-GlcNAcylation levels in several pathologies and, consequently, prevents adverse effects. At contrary, O-GlcNAcylation of AMPK observed in different diseases reduces its capacity to play its beneficial role, contributing to the progression of the disease. This highlights AMPK and O-GlcNAcylation crosstalk as novel putative therapeutic target for important diseases such as cancer, diabetes and cardiovascular diseases. However, additional studies are still required to fully unravel the complexity of the relationship between AMPK and O-GlcNAcylation with respect to these diseases.

## Author contributions

RG, JD, LaB, and LuB wrote sections of the first draft of the manuscript. JD realized the figures. RG, SH, CB, LaB, and LuB participated in the fusion of the global version. All authors contributed to manuscript revision, read and approved the submitted version.

### Conflict of interest statement

The authors declare that the research was conducted in the absence of any commercial or financial relationships that could be construed as a potential conflict of interest.
